# Nutrition, T-2 Toxin, and Gut Microbiota in Kashin–Beck Disease: Current Evidence and Proposed Links Within the Microbiota–Gut–Osteochondral Framework

**DOI:** 10.3390/metabo16100787

**Published:** 2026-10-16

**Authors:** Yang Liu, Chaowei Wang, Liangyu Cui, Xiong Guo, Xi Wang, Yujie Ning

**Affiliations:** 1 Department of Occupational and Environmental Health, School of Public Health, Health Science Center, Xi’an Jiaotong University, Xi’an 710061, China; lyang1226@stu.xjtu.edu.cn (Y.L.); cuiliangyu@stu.xjtu.edu.cn (L.C.); 2 Collaborative Innovation Center of Endemic Disease and Health Promotion for Silk Road Region, School of Public Health, Health Science Center, Xi’an Jiaotong University, Xi’an 710061, China; wcw5205060@163.com (C.W.); guox@xjtu.edu.cn (X.G.); 3 Key Laboratory of Trace Elements and Endemic Diseases, National Health and Family Planning Commission, School of Public Health, Health Science Center, Xi’an Jiaotong University, Xi’an 710061, China

**Keywords:** Kashin–Beck disease, dietary factors, T-2 toxin, gut microbiota, microbiota–gut–osteochondral framework

## Abstract

**Highlights:**

**What are the main findings?**

- Human studies have identified KBD-associated alterations in gut microbial composition and metabolic profiles, whereas experimental models support the osteochondral effects of selenium deficiency and T-2 toxin exposure.
- A microbiota–gut–osteochondral framework may provide a biological context linking environmental exposures, host nutritional status, and cartilage injury, although most mechanistic connections remain to be experimentally validated in KBD.

**What are the implications of the main findings?**

- An evidence-stratified framework helps distinguish direct KBD observations, KBD-related experimental evidence, and hypotheses derived from broader microbiome and skeletal research.
- Future longitudinal and mechanistic studies integrating microbiome, metabolome, immune profiling, and nutritional assessment are required to clarify causal relationships and evaluate the potential of microbiota- and nutrition-based approaches.

**Abstract:**

Kashin–Beck disease (KBD) is an endemic osteoarthropathy characterized by the degeneration and necrosis of articular cartilage and epiphyseal growth plate cartilage, with disease onset occurring predominantly during childhood. Its etiology has long been associated with environmental and nutritional factors, particularly selenium deficiency, T-2 toxin exposure, and dietary imbalance. Recent studies have also reported alterations in gut microbial composition and metabolic profiles in patients with KBD, raising the possibility that the gut microbiota may represent an additional component of its multifactorial etiology. This narrative review critically evaluates current evidence linking nutritional factors, T-2 toxin exposure, gut microbial alterations, and osteochondral injury in KBD, with particular attention to the level of evidence supporting these relationships. Direct human evidence is currently limited to a small number of predominantly cross-sectional microbiome and metabolomic studies, largely involving adults with established KBD. KBD-related experimental models provide evidence that selenium deficiency and T-2 toxin exposure can induce osteochondral injury, including combined-exposure effects, and experimental studies also indicate that these exposures can be associated with alterations in gut microbial composition. However, these findings do not demonstrate that gut microbial changes mediate exposure-related osteochondral injury. Proposed pathways involving short-chain fatty acids (SCFAs), intestinal-barrier dysfunction, lipopolysaccharide (LPS)-related inflammatory signaling, immune regulation, and bone remodeling are supported mainly by experimental or mechanistic evidence from non-KBD settings and have not been validated as an integrated causal pathway in KBD. The microbiota–gut–osteochondral framework should therefore be regarded as an evidence-stratified, hypothesis-generating model. Prospective studies in children and adolescents from endemic areas, together with factorial exposure models, direct measurement of proposed intermediate mediators, and functional microbiota-manipulation experiments, are needed to determine whether gut microbial alterations contribute causally to KBD development.

## 1. Introduction

Kashin–Beck disease (KBD) is a chronic endemic osteoarthropathy that primarily affects children and adolescents. Pathologically, it is characterized by degeneration and necrosis of articular cartilage and epiphyseal growth plate cartilage [1]. KBD is predominantly distributed along a broad endemic belt extending from northeastern to southwestern China, with additional endemic regions historically reported in eastern Siberia, Russia, and North Korea [2]. Despite decades of comprehensive prevention and control efforts that have significantly reduced the incidence of new KBD cases, individuals affected by the disease continue to suffer from irreversible skeletal deformities, impaired physical function, and a diminished quality of life. Consequently, KBD remains a significant public health concern and serves as a unique model for studying environmentally associated osteochondral disorders.

For several decades, etiological research on KBD has focused on selenium deficiency, mycotoxin exposure, and nutritional insufficiency. Selenium deficiency, contamination of staple grains with Fusarium-derived mycotoxins—particularly T-2 toxin—and inadequate intake of vitamins, proteins, and essential trace elements have been proposed as important risk factors for the development and progression of disease [3,4]. These factors may contribute to cartilage damage through mechanisms such as oxidative stress [5], mitochondrial dysfunction [6], chondrocyte apoptosis [7], and extracellular matrix degradation [8]. However, no single etiological hypothesis adequately accounts for the pronounced geographic clustering of KBD, the substantial interindividual variation in disease susceptibility, or its heterogeneous pathological manifestations. These observations imply that KBD is more likely the result of intricate interactions among environmental exposures, nutritional status, host genetic susceptibility, and endogenous regulatory mechanisms.

In recent years, the gut microbiota has emerged as a potential area of investigation for understanding the multifactorial context of KBD. Beyond its roles in nutrient metabolism and energy extraction, the gut microbiota can influence immune and inflammatory responses and skeletal homeostasis through microbial metabolites and components, including short-chain fatty acids (SCFAs), bile acid derivatives, and lipopolysaccharide (LPS) [9,10,11]. Studies of osteoporosis, osteoarthritis (OA), rheumatoid arthritis, and other inflammatory disorders have increasingly implicated gut microbial dysbiosis in altered intestinal barrier integrity, systemic immune regulation, and skeletal metabolism [12,13,14]. However, these findings are derived largely from non-KBD settings and therefore provide biological plausibility rather than direct evidence of KBD-specific mechanisms. Recent studies have begun to examine the potential involvement of the gut microbiota in KBD more directly. Wu et al. reported alterations in gut microbial composition in rats subjected to selenium deficiency or T-2 toxin exposure, together with osteochondral abnormalities, providing KBD-related experimental evidence linking these environmental factors with both microbial and cartilage changes [15]. More recently, a hypothesis-focused article proposed a “cartilage–gut–microbiome” axis linking selenium deficiency and T-2 toxin exposure to KBD [16]. Rather than proposing a similar mechanistic hypothesis, the present review focuses on critically evaluating the strength of evidence supporting the individual links within this framework.

**Figure 1:**
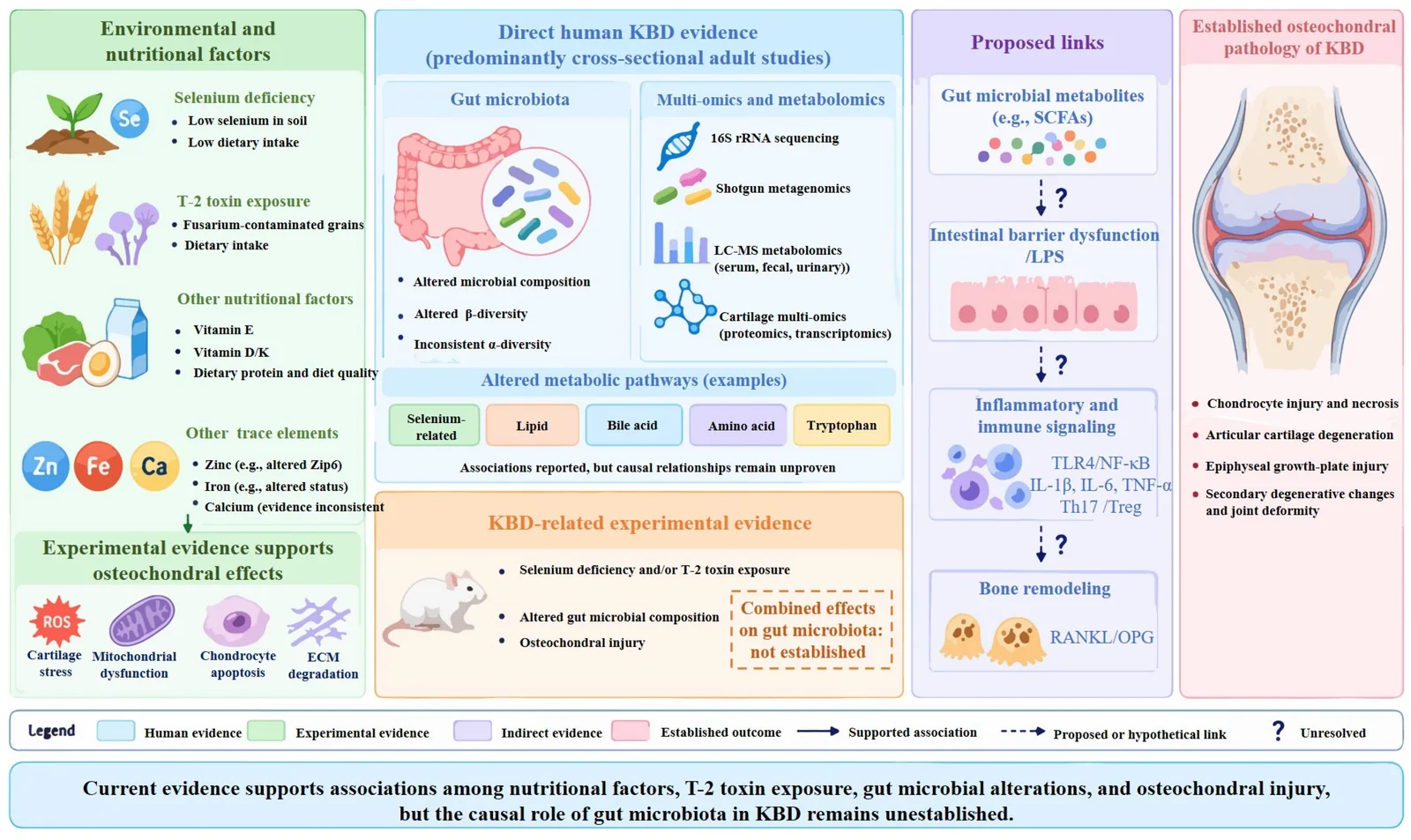
Overview of current evidence linking nutritional factors, T-2 toxin exposure, gut microbial alterations, and osteochondral injury in KBD (KBD, Kashin–Beck disease, SCFAs: short-chain fatty acids, LPS: lipopolysaccharide, RANKL: receptor activator of nuclear factor-κB ligand, OPG: osteoprotegerin, TLR4: Toll-like receptor 4, NF-κB: nuclear factor kappa B, IL-1β: interleukin-1 beta, IL-6: interleukin-6, TNF-α: tumor necrosis factor alpha, Th17: T helper 17 cells, ECM: extracellular matrix).

Available human studies have reported differences in gut microbial composition and metabolomic profiles between patients with KBD and comparator groups [17,18,19]. However, these findings are based on a small number of predominantly cross-sectional studies, largely involving adults with established disease, whereas KBD typically begins during childhood. Against this background, the present narrative review critically evaluates evidence linking nutritional factors, particularly selenium deficiency, T-2 toxin exposure, gut microbial alterations, and osteochondral injury in KBD. Evidence from human KBD studies, KBD-related animal models, in vitro studies, and mechanistic research in other disease settings is considered separately, with particular attention to distinguishing experimentally supported findings from proposed or hypothesized links. By integrating these different levels of evidence, we aim to clarify which components of the proposed microbiota–gut–osteochondral framework are directly supported and which remain insufficiently validated, while identifying key priorities for future research. Figure 1 summarizes the current evidence linking nutritional factors, T-2 toxin exposure, gut microbial alterations, and osteochondral injury in KBD.

## 2. Materials and Methods

This article was designed as a narrative review to critically evaluate evidence linking nutritional factors, T-2 toxin exposure, gut microbial alterations, and osteochondral injury in KBD. Relevant literature was identified through searches of PubMed/MEDLINE, Web of Science Core Collection, China National Knowledge Infrastructure (CNKI), and Wanfang Data from database inception to 1 September 2026. The search was organized around the major themes of this review, including KBD in combination with gut microbiota or microbiome, selenium and other nutritional factors, T-2 toxin and other mycotoxins, metagenomics and metabolomics, intestinal-barrier function, short-chain fatty acids, lipopolysaccharide-related signaling, immune regulation, and osteochondral injury. Corresponding Chinese search terms were used in CNKI and Wanfang Data. Articles published in English or Chinese were considered. Reference lists of relevant reviews and primary studies were also examined to identify additional publications.

Priority was given to original studies directly involving patients with KBD or experimental models relevant to KBD. Human studies from KBD-endemic populations, KBD-related animal studies involving selenium deficiency, T-2 toxin or combined exposures, and relevant in vitro studies were considered when they addressed the scope of this review. Because direct mechanistic evidence in KBD is limited, non-KBD experimental and clinical studies were included selectively when they provided biological context for specific proposed pathways. Duplicate publications, studies unrelated to KBD or the proposed framework, and non-KBD studies without direct relevance to the pathways discussed were excluded from detailed consideration.

To distinguish direct observations from mechanistic extrapolation, evidence was categorized as (1) direct human KBD evidence; (2) KBD-related animal evidence; (3) in vitro evidence; and (4) indirect mechanistic evidence derived from other diseases or general microbiome research. The literature search was intended to support a critical narrative synthesis rather than a quantitative systematic review or meta-analysis; accordingly, mechanistic pathways supported only by indirect evidence are described as proposed or biologically plausible rather than as established KBD-specific mechanisms. Table 1 summarizes the key findings and limitations of studies linking nutritional factors, T-2 toxin exposure, gut microbiota alterations, and osteochondral injury in KBD. Detailed study information is provided in Table A1.

## 3. Dietary Nutritional Factors Associated with KBD

### 3.1. Selenium Deficiency and Selenoprotein Dysfunction

Selenium is an essential trace element whose biological functions are largely mediated by selenoproteins involved in redox regulation and antioxidant defense [28,29,30]. Key selenoproteins, such as glutathione peroxidases, thioredoxin reductases, and selenoprotein P, contribute to the control of reactive oxygen species (ROS), maintenance of cellular redox homeostasis, and protection against oxidative injury [28,31]. Given the susceptibility of chondrocytes to oxidative stress, impairment of selenium-dependent antioxidant defense may increase oxidative damage and thereby enhance the vulnerability of osteochondral tissues to injury [32,33].

KBD has historically occurred in regions characterized by low environmental selenium availability, and epidemiological studies have reported lower selenium status in populations from endemic areas [3,34,35]. A recent survey across historically severe KBD-endemic areas showed that low selenium levels remain common in staple grains despite generally moderate selenium status in children, highlighting persistent heterogeneity in selenium exposure across endemic regions [36]. Experimental evidence suggests that selenium deficiency may disrupt selenoprotein expression and impair antioxidant defenses, thereby increasing oxidative stress and promoting chondrocyte apoptosis [32]. At the molecular level, selenium deficiency has been linked to altered runt-related transcription factor 2 (*RUNX2*) methylation and expression in KBD chondrocytes [37]. However, selenium deficiency alone does not reproduce the full epidemiological and pathological characteristics of KBD, and not all individuals living in selenium-deficient areas develop the disease.

Selenium deficiency should therefore be regarded as an important predisposing or susceptibility factor, rather than an independently sufficient cause of KBD. Its contribution is likely to depend on interactions with other environmental exposures, nutritional conditions, and host susceptibility. Evidence that selenium deficiency contributes to KBD specifically through alterations of the gut microbiota remains limited.

### 3.2. Other Nutritional Factors Potentially Relevant to KBD

In addition to selenium, other nutritional factors have been investigated as potential modifiers of KBD susceptibility. Vitamin E is of particular interest because of its antioxidant properties and its functional relationship with selenium-dependent antioxidant systems. Experimental evidence indicates that inadequate antioxidant protection can increase cellular susceptibility to oxidative injury, whereas selenium and vitamin E may exert complementary protective effects against oxidative stress [38]. However, the independent contribution of vitamin E deficiency to KBD in human populations remains insufficiently established. Vitamins D and K also contribute to skeletal homeostasis through their roles in calcium metabolism and bone health [39]. Nevertheless, evidence directly linking deficiencies of these vitamins to the initiation of KBD is limited. Notably, calcium and/or vitamin D supplementation did not improve growth, KBD, or bone-metabolism outcomes in a prospective study of children living in a KBD-endemic area of Tibet [40]. These findings suggest that the roles of calcium and vitamin D in KBD are likely more complex than a simple deficiency model. Associations identified in endemic populations should therefore be interpreted cautiously because nutritional status may covary with age, geographic location, dietary patterns, socioeconomic conditions, and other environmental exposures.

Dietary studies conducted in KBD-endemic areas have also suggested that nutrients other than selenium may contribute to the nutritional context in which KBD occurs [41]. However, current evidence does not justify attributing KBD to generalized protein–energy malnutrition or dietary monotony alone. Rather, overall dietary quality and multiple nutritional factors may modify susceptibility to osteochondral injury in combination with selenium status and environmental exposures. Whether these nutritional factors influence KBD through alterations of the gut microbiota remains largely unexplored.

### 3.3. Other Trace Element Imbalance and Osteochondral Metabolic Dysfunction

In addition to selenium, other trace elements, particularly zinc and iron, have been investigated in relation to KBD, although their specific contributions remain incompletely understood. Zinc is involved in chondrocyte function, cartilage development, and extracellular matrix homeostasis [42,43]. In KBD-endemic regions, regional variation in zinc exposure has been documented through assessments of dietary and drinking-water intake [44], while differences in zinc status among children have also been reported based on trace element measurements in biological samples, such as scalp hair, across endemic areas [45]. Wu et al. further reported alterations in Zrt- and Irt-like protein 6 (ZIP6) expression and Zn^2+^ homeostasis in adult and pediatric KBD chondrocytes and in experimental models involving selenium deficiency and T-2 toxin exposure, together with changes in extracellular-matrix-related genes [24]. Iron status may also be relevant to KBD, although current evidence is inconsistent. Higher hair iron concentrations have been reported in children from KBD-endemic areas, particularly in children with KBD, compared with non-endemic controls [46]; however, a meta-analysis did not identify consistent differences in hair or serum iron between KBD patients and healthy controls [47]. Given that excessive labile iron can promote ROS generation and oxidative damage, the potential involvement of iron in KBD-associated oxidative stress warrants further investigation [48]. Overall, the contribution of individual trace elements to KBD remains uncertain and may vary with regional dietary patterns, environmental exposure, age, nutritional status, and the biological samples examined.

## 4. T-2 Toxin Exposure, Nutritional Status, and Proposed Gut Links

### 4.1. T-2 Toxin and Osteochondral or Intestinal Injury

T-2 toxin is a type A trichothecene mycotoxin produced by *Fusarium* species and has been investigated as an environmental risk factor for KBD [49]. Previous studies have demonstrated cytotoxic effects of T-2 toxin, including inhibition of protein synthesis, oxidative stress, mitochondrial dysfunction, and apoptosis [6,49,50,51]. KBD-related studies further support direct toxic effects on chondrocytes and osteochondral tissues [7]. T-2 toxin has been shown to compromise intestinal epithelial barrier integrity, disrupt tight-junction proteins, and perturb cellular redox homeostasis [25,52,53], and has also been associated with alterations in gut microbiota in experimental models [54,55]. However, these findings demonstrate toxic effects on cartilage and the intestine but do not establish that intestinal injury or gut microbial alterations mediate T-2 toxin-induced osteochondral damage. In particular, the downstream sequence involving intestinal-barrier dysfunction, LPS translocation, and systemic inflammatory signaling has not been directly demonstrated in KBD-related models and should therefore be considered a proposed pathway rather than an established mechanism.

### 4.2. Selenium Status and T-2 Toxin-Related Osteochondral Injury

Experimental studies using KBD-related models indicate that selenium deficiency can modify the osteochondral response to T-2 toxin exposure. Combined selenium-deficiency and T-2 toxin models have reproduced characteristic cartilage abnormalities, including chondronecrosis and epiphyseal growth-plate lesions [23,56]. Other studies have further demonstrated increased chondrocyte apoptosis and disturbances in cartilage matrix homeostasis under these exposure conditions [7,23]. These findings suggest that selenium status influences tissue susceptibility to T-2 toxin rather than acting as an independently sufficient determinant of cartilage injury.

Mechanistic studies have further identified several cellular processes through which selenium deficiency and T-2 toxin exposure, alone or in combination, may contribute to osteochondral injury. Sun et al. showed that both selenium deficiency and T-2 toxin exposure impaired SECIS-binding protein 2 (SECISBP2)-mediated selenoprotein expression and cartilage homeostasis, with more pronounced abnormalities under combined exposure [23]. Zhou et al. showed altered type II collagen together with dysregulation of matrix metalloproteinase 1 (MMP1), matrix metalloproteinase 3 (MMP3), matrix metalloproteinase 13 (MMP13), and tissue inhibitor of metalloproteinases 1 (TIMP1) in a low-selenium rat model exposed to T-2 toxin, supporting disruption of extracellular matrix turnover under combined exposure conditions [57]. In addition, Deng et al. identified altered chondrocyte autophagy and AMP-activated protein kinase (AMPK)/mechanistic target of rapamycin (mTOR)/Unc-51-like kinase 1 (ULK1) signaling in KBD cartilage and in rats exposed to low selenium, T-2 toxin, or their combination [4]. SIRT1 (sirtuin 1) hypermethylation and reduced expression were also observed in KBD cartilage and were associated with increased chondrocyte apoptosis; similar changes were induced by selenium deficiency or T-2 toxin and were partially reversed by selenium supplementation [58]. Taken together, these findings suggest that selenium deficiency and T-2 toxin exposure may disrupt multiple processes involved in osteochondral homeostasis, including selenoprotein function, extracellular-matrix maintenance, autophagy, and epigenetic regulation.

### 4.3. Potential Links Among T-2 Toxin, Selenium Deficiency, and the Gut Microbiota

Wu et al. examined rats exposed to selenium deficiency or T-2 toxin and reported osteochondral abnormalities together with exposure-associated differences in gut microbial composition [15]. Notably, the microbial patterns associated with selenium deficiency and T-2 toxin were not identical, suggesting that these environmental exposures may exert distinct effects on the intestinal microbial ecosystem.

The relationship between these exposure-associated microbial alterations and osteochondral injury remains unclear. Although selenium deficiency and T-2 toxin have been studied together in KBD-related osteochondral models [5,7,23,56], their combined effects on the gut microbiota have not been established. Current evidence therefore supports potential links among nutritional status, T-2 toxin exposure, and gut microbial alterations, but the nature and biological significance of these relationships in KBD require further investigation.

## 5. Gut Microbiota Characteristics in Patients with KBD

### 5.1. Alterations in Gut Microbial Diversity and Composition

Available human studies have identified differences in gut microbial diversity and composition between patients with KBD and comparator groups, although the findings vary according to the sequencing approach and comparator population. In a study of 32 patients with KBD and 35 healthy controls, 16S rDNA sequencing showed no significant differences in alpha-diversity indices, including Shannon, observed-species, and Chao1, whereas beta-diversity analyses revealed significant differences in microbial community structure between the two groups [17]. Taxonomic analysis further identified differences at both the phylum and genus levels, including higher relative abundances of Bacteroidota and Fusobacteriota and lower relative abundance of Firmicutes in the KBD group, together with a number of differentially abundant genera [17]. In contrast to the 16S results, shotgun metagenomic analysis of the same participants showed significantly lower alpha diversity in both grade I and grade II KBD groups than in healthy controls and further identified differences in microbial composition at the species level [17].

Ning et al. subsequently compared patients with KBD and patients with OA and reported differences in gut microbial composition, including higher relative abundances of *Prevotella_9*, *Lactobacillus*, and *Coprococcus_2* in the KBD group [18]. Notably, the KBD participants overlapped with those in Wang et al. [17]; therefore, the two studies should not be considered independent replication cohorts. Overall, the available human studies suggest KBD-associated alterations in gut microbial composition, whereas evidence for changes in microbial diversity varies according to the sequencing approach and comparator population. Given the limited number of independent cohorts, a consistent KBD-specific microbial signature has yet to be confirmed.

An important limitation of the available human microbiome evidence is the disease stage at which participants have been studied. KBD is typically initiated during childhood, when lesions develop in articular and epiphyseal growth-plate cartilage, whereas the currently available gut microbiome and fecal metabolomics studies have largely examined adults with established KBD. For example, the fecal metabolomics study by Wen et al. included patients with a mean age of approximately 59 years [19]. Thus, microbial or metabolic differences detected in these populations may characterize adult residual KBD rather than biological events involved in disease initiation. Age-related changes in the gut microbiota, reduced mobility, medication or supplement use, long-term dietary adaptation, chronic pain, and secondary degenerative joint changes may all contribute to the observed profiles [59,60,61]. Reverse causation and residual confounding should therefore be considered when interpreting KBD-associated microbial differences.

### 5.2. Functional and Metabolic Alterations Associated with KBD

Beyond differences in microbial composition, multi-omics studies have identified alterations in microbial functional potential and metabolic profiles associated with KBD. In the study by Wang et al. [17], Phylogenetic Investigation of Communities by Reconstruction of Unobserved States 2 (PICRUSt2) analysis based on 16S rDNA sequencing identified differences in predicted microbial functional potential between patients with KBD and healthy controls. Shotgun metagenomic analysis further revealed differences in microbial gene abundance and pathway profiles among grade I KBD, grade II KBD, and healthy control groups, involving carbohydrate, sphingolipid, glutathione, and vitamin B6 metabolism [17]. These findings indicate alterations in microbial functional potential but do not directly demonstrate changes in gene expression or metabolite production.

Serum liquid chromatography–mass spectrometry (LC-MS) metabolomics further revealed alterations primarily involving lipid-related pathways, including unsaturated fatty acid and glycerophospholipid metabolism [17]. Several unsaturated fatty acids, including docosapentaenoic acid, γ-linolenic acid, and oleic acid, were negatively correlated with *Paenibacillus montanisoli* and positively correlated with *Buttiauxella brennerae* [17], suggesting potential microbiome–metabolome associations. Untargeted fecal ultra-high-performance liquid chromatography–mass spectrometry metabolomics identified alterations in selenium-related, lipid, bile acid, and amino acid metabolic pathways in patients with KBD [19]. A urinary LC-MS metabolomics study further reported alterations in several metabolic pathways, including arachidonic acid and amino acid metabolism [22]. Beyond metabolomic evidence, multi-omics profiling of KBD cartilage identified alterations in proteins and transcripts associated with metabolic processes, including enrichment of tryptophan metabolism at the proteomic level [20]. Collectively, these findings support the presence of metabolic disturbances associated with KBD, although their causal relevance remains unclear.

Nevertheless, predicted microbial functional potential, metagenomic gene abundance, and measured metabolites represent distinct levels of evidence. Moreover, microbiome–metabolome correlations do not establish causality, and measured metabolites cannot be assumed to be microbiota-derived without direct evidence of their microbial origin. Direct functional links connecting gut microbial alterations, metabolite production, and osteochondral injury in KBD therefore remain to be established.

### 5.3. Interpretation According to Age, Disease Stage, and Residual Degenerative Changes

Human KBD microbiome findings should be interpreted in the context of disease stage. Juvenile active KBD, adult residual KBD, and secondary osteoarthritic changes represent biologically distinct settings. Juvenile KBD reflects the period in which growth plate and articular cartilage lesions develop, whereas adult residual KBD represents the long-term structural consequences of disease acquired earlier in life [1]. In adults, secondary osteoarthritic remodeling, chronic inflammation, altered mobility, and treatment-related factors may further influence both musculoskeletal and microbial phenotypes.

Consequently, gut microbial differences observed in adults with KBD cannot be assumed to represent initiating factors in childhood disease. They may instead represent persistent correlates of the disease, consequences of long-standing disability, secondary osteoarthritic changes, or age-related host–microbiome alterations. Prospective studies in children and adolescents from endemic areas are therefore required to determine whether microbial changes precede or follow osteochondral injury.

## 6. Dietary Factors and Gut Microbiota

### 6.1. Selenium and Gut Microbiota

Experimental evidence suggests that selenium availability can influence the intestinal microbial environment. In a KBD-related rat model, selenium deficiency was associated with alterations in gut microbial composition [15]. Outside the KBD context, dietary selenium has also been shown to alter gut microbial composition and host selenoproteome expression in experimental models [27]. Studies using selenium-containing dietary components have also reported alterations in gut microbial composition and intestinal inflammatory phenotypes in experimental disease models [62,63]. These findings suggest that selenium status or selenium-containing dietary components can influence the gut microbial ecosystem, although the effects may depend on selenium form, dose, host status, and experimental context.

These observations provide biological plausibility for a selenium–microbiota relationship but do not establish that gut microbial alterations mediate the effects of selenium deficiency in KBD. Direct human KBD studies simultaneously assessing selenium status, gut microbiota, and osteochondral outcomes are currently lacking.

### 6.2. Dietary Fiber and Short-Chain Fatty Acid Production

Dietary fiber is fermented by intestinal microorganisms to generate SCFAs, principally acetate, propionate, and butyrate [64]. SCFAs, particularly butyrate, contribute to intestinal epithelial homeostasis and represent important mediators of diet–microbiota interactions [65]. However, direct evidence linking dietary fiber intake to measured SCFA concentrations in patients with KBD is scarce, and reduced SCFA production has not been established as a characteristic metabolic feature of KBD. Further studies integrating dietary assessment with direct measurement of SCFAs are needed to clarify whether fiber-related microbial metabolism is altered in KBD.

### 6.3. Vitamin D and the Gut Microbiota

Vitamin D has been associated with gut microbial composition and intestinal homeostasis in experimental studies outside KBD [66]. Human intervention studies further suggest that vitamin D supplementation can modify gut microbial composition [67,68,69]. In vitamin-D-deficient healthy women, vitamin D supplementation was associated with changes in microbial diversity and the relative abundance of several bacterial taxa [67]. A randomized dose–response trial in healthy adults also reported changes in gut microbial composition following 8 weeks of vitamin D3 supplementation [68]. However, the reported microbial responses vary across study populations, vitamin D doses, and experimental settings, and a consistent vitamin D-associated microbial signature has not been established.

In KBD, evidence directly linking vitamin D status, gut microbial alterations, and osteochondral injury is currently lacking. Thus, although vitamin D may represent another nutritional factor capable of modifying the intestinal microbial environment, its relevance to the proposed microbiota–gut–osteochondral framework in KBD remains to be determined.

## 7. Proposed Microbiota–Gut–Osteochondral Pathways in KBD

### 7.1. Evidence-Stratified Microbiota–Gut–Osteochondral Framework

The evidence summarized in Table 1 indicates that direct KBD studies have mainly demonstrated alterations in gut microbial profiles, metabolic signatures, and osteochondral changes, whereas mechanistic links connecting these observations remain incompletely established. The gut microbiota has been implicated in skeletal homeostasis through microbial metabolism, intestinal barrier function, and immune regulation [70,71]. These relationships provide a biological basis for considering a microbiota–gut–osteochondral framework in KBD. However, most mechanistic evidence underlying this framework derives from general microbiome research or studies of other skeletal disorders rather than from KBD itself. The framework should therefore be interpreted as a set of biologically plausible links rather than an established causal cascade in KBD.

Available evidence can be considered at different levels. Human KBD studies provide direct evidence of differences in gut microbial composition, microbial functional potential, and metabolic profiles [17,18,19]. KBD-related experimental studies indicate that selenium deficiency and T-2 toxin exposure can induce osteochondral injury and may also be associated with alterations in gut microbial composition [7,15]. Combined selenium-deficiency and T-2 toxin models further support osteochondral effects under combined exposure conditions. However, current evidence does not establish that gut microbial alterations mediate these exposure-related osteochondral effects.

**Figure 2:**
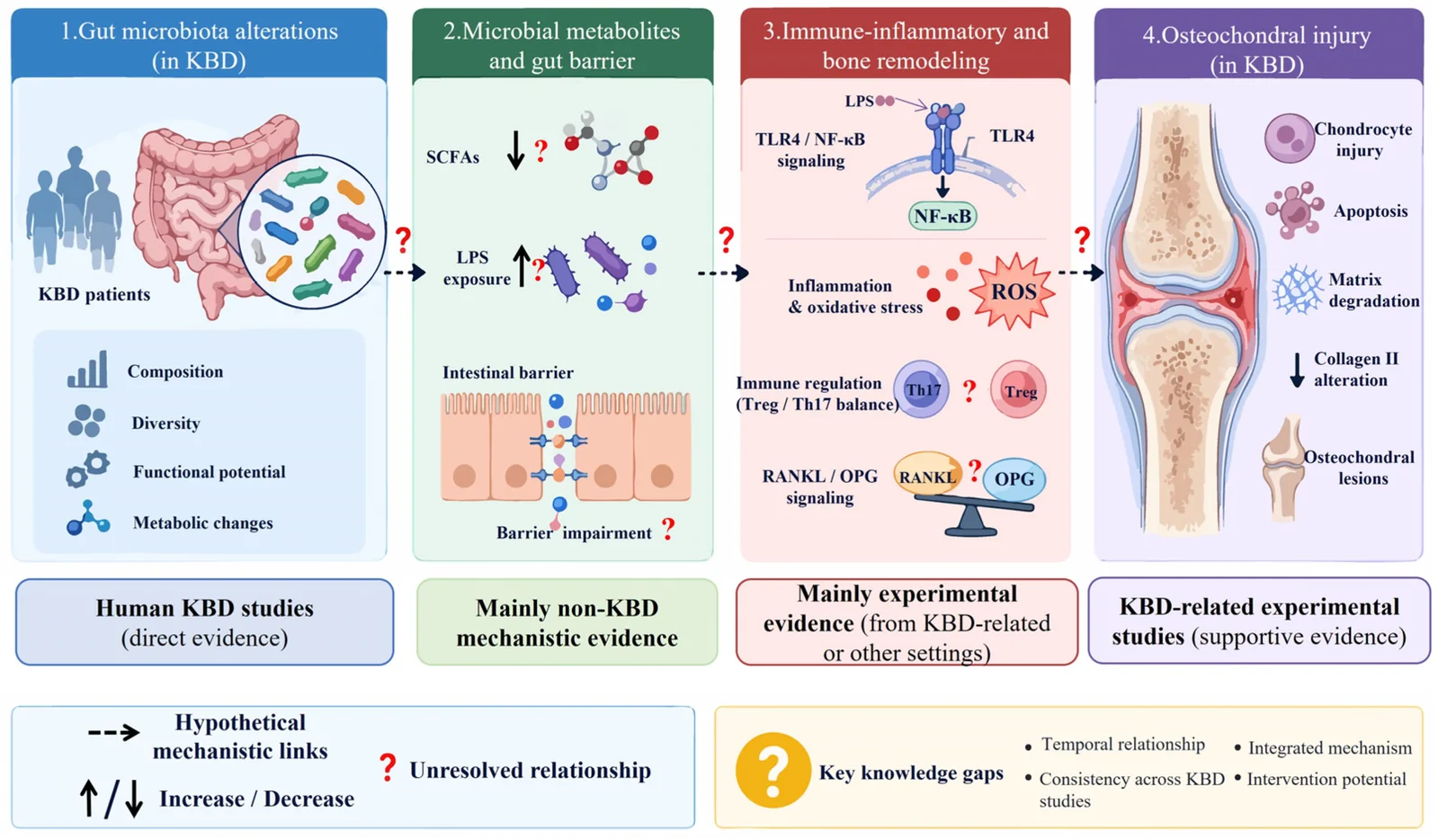
Evidence-stratified conceptual framework of proposed microbiota–gut–osteochondral pathways in KBD (SCFAs: short-chain fatty acids, LPS: lipopolysaccharide, TLR4: Toll-like receptor 4, NF-κB: nuclear factor kappa B, ROS: reactive oxygen species, Treg: regulatory T cells, Th17: T helper 17 cells, RANKL: receptor activator of nuclear factor-κB ligand, OPG: osteoprotegerin).

Accordingly, Figure 2 presents an evidence-stratified conceptual framework rather than an established mechanistic cascade. The proposed links involve microbial metabolites, intestinal barrier function, inflammatory and immune signaling, and osteochondral homeostasis, but these components are supported by different levels of evidence and have not been validated as an integrated pathway in KBD.

### 7.2. SCFAs and Intestinal Barrier Function

SCFAs, predominantly acetate, propionate, and butyrate, are microbial metabolites produced through the fermentation of dietary fiber [64]. Experimental studies outside KBD have shown that SCFAs can influence intestinal epithelial integrity, immune regulation, and bone metabolism [11,65,72,73]. In vitro, butyrate has been shown to facilitate tight-junction assembly and enhance epithelial barrier function in Caco-2 cell monolayers [73]. In experimental models, propionate and butyrate have been shown to suppress osteoclast differentiation and bone resorption [11], while butyrate has also been linked to regulatory T-cell-mediated osteoimmune regulation [72].

These findings provide biological plausibility for a potential SCFA-related link between gut microbial metabolism and osteochondral homeostasis. However, current human KBD studies have not established reduced fecal or circulating SCFA concentrations as a reproducible disease feature, nor have they demonstrated a linked relationship among SCFA alterations, intestinal permeability, immune changes, and osteochondral injury [17,18,19]. Therefore, an SCFA-mediated gut–osteochondral pathway in KBD should currently be regarded as a proposed link rather than an established mechanism.

### 7.3. LPS–TLR4/NF-κB Signaling and Osteochondral Injury

Impairment of the intestinal barrier can facilitate systemic exposure to microbial components such as LPS in experimental and non-KBD settings [74]. LPS can be recognized by TLR4 (Toll-like receptor 4) and activate downstream nuclear factor kappa B (NF-κB) signaling, promoting the production of pro-inflammatory cytokines, including IL-1β (interleukin-1β), IL-6 (interleukin-6), and TNF-α (tumor necrosis factor-α) [75,76]. These findings provide biological plausibility for a potential link between intestinal microbial alterations, systemic inflammation, and osteochondral homeostasis. However, increased intestinal permeability, circulating LPS, or gut-derived TLR4 activation has not been directly demonstrated in KBD.

Several downstream inflammatory and bone-remodeling components relevant to this proposed pathway have been examined in KBD or KBD-related models. NF-κB-p65 and receptor activator of nuclear factor-κB ligand (RANKL) expression have been investigated in articular cartilage from patients with KBD, with age-dependent alterations reported between juvenile and adult disease [21]. Altered circulating RANKL and osteoprotegerin (OPG) levels have also been reported in KBD-related experimental models [77]. Experimental evidence outside KBD further supports roles for LPS–TLR4 signaling, inflammatory cytokines, and T helper 17 (Th17)/regulatory T (Treg) cell balance in osteoimmune regulation [26,78,79]. However, these findings have not been directly linked to gut-derived signals in KBD.

Importantly, these inflammatory and bone-remodeling changes may represent complementary processes associated with disease progression, tissue repair, and remodeling, rather than substitutes for the hallmark pathological features of KBD, which are characterized primarily by chondrocyte injury and necrosis in the articular and epiphyseal growth-plate cartilage [3,80]. Accordingly, although individual components of the proposed pathway are biologically plausible or supported by experimental evidence, a sequential link from gut dysbiosis and intestinal-barrier dysfunction to LPS/TLR4/NF-κB signaling, immune dysregulation, and osteochondral injury has not been established in KBD.

## 8. Research Gaps and Future Directions

### 8.1. Establishing Causality and Disease-Stage Relevance

Current human microbiome studies in KBD are predominantly cross-sectional and largely involve adults with established disease [17,18,19]. Because KBD originates in childhood and affects the growth plate and articular cartilage [1], adult microbial profiles may not fully represent the microbial state during disease initiation [81,82]. Adult microbiome findings may reflect aging, reduced mobility, medication use, dietary adaptation, selenium supplementation, chronic pain, or secondary OA. Reverse causation and residual confounding therefore remain major concerns.

Prospective studies should prioritize children and adolescents in endemic areas and distinguish among active juvenile KBD, adult residual KBD, and secondary osteoarthritic changes in adults with KBD. Repeated assessment of nutritional status, T-2 toxin exposure, gut microbial profiles, metabolic markers, and imaging during the period of disease risk would help determine whether microbial alterations precede osteochondral injury or emerge as consequences of established disease. Adult case–control studies should additionally account for age, mobility, medication use, nutritional supplementation, chronic pain, diet, and coexisting OA wherever possible.

Factorial designs incorporating selenium-replete and selenium-deficient conditions with and without T-2 toxin are needed to distinguish independent, additive, and formal interaction effects. Germ-free, antibiotic-treated, defined-colonization, or microbiota-transfer approaches could subsequently determine whether microbial changes mediate or modify exposure-related osteochondral injury.

### 8.2. Functional Validation of Microbiota-Related Mechanisms

Future studies should integrate shotgun metagenomics with metatranscriptomics, targeted metabolomics, host molecular profiling, and clinical phenotyping to distinguish microbial functional potential from actual microbial activity and host metabolic responses. Longitudinal sampling would allow microbial gene abundance, transcript abundance, measured metabolites, inflammatory markers, and osteochondral phenotypes to be evaluated in temporal sequence.

Particular priority should be given to direct measurement of currently proposed intermediate components, including fecal and circulating SCFAs, intestinal permeability, circulating LPS, relevant inflammatory signaling, and immune-cell profiles. Predicted pathways and taxon–metabolite correlations should subsequently undergo functional validation using microbial manipulation, metabolite supplementation or depletion, or pathway-specific experiments.

### 8.3. Potential Interventions and Translational Considerations

Nutritional and microbiota-targeted approaches should currently be regarded as research possibilities rather than established treatments for KBD. Selenium intervention has historically been relevant to KBD prevention, particularly in selenium-deficient populations [3]. However, prevention in children at risk should be distinguished from treatment of adults with established KBD. Future intervention studies should consider baseline selenium status, dose, chemical form, age, disease stage, and safety limits because selenium has a relatively narrow range between nutritional adequacy and excessive exposure [83].

Dietary fiber, probiotics, prebiotics, and synbiotics may provide experimental approaches for testing whether modification of the gut microbiota or microbial metabolites influences KBD-related outcomes. However, no KBD-specific clinical trial has demonstrated therapeutic efficacy of microbiota-targeted interventions. These approaches should therefore first be evaluated for their ability to reproducibly modify microbial or metabolic features and experimentally defined osteochondral outcomes.

Fecal microbiota transplantation is particularly speculative in KBD and should currently be regarded primarily as an experimental tool for testing microbial causality rather than as a therapeutic option. Microbiota-transfer studies in appropriate animal models could help determine whether KBD-associated microbial communities modify susceptibility to selenium deficiency or T-2 toxin-related osteochondral injury.

## 9. Conclusions

Current evidence supports a multifactorial view of KBD involving nutritional status, environmental exposures, host susceptibility, and potentially the gut microbiota. However, the relationships among these factors are supported by different levels of evidence. Direct human KBD evidence is currently limited to a small number of predominantly cross-sectional studies showing differences in gut microbial composition, microbial functional potential, and metabolic profiles, largely in adults with established disease. These findings support KBD-associated microbial and metabolic alterations but do not establish their temporal or causal relationship with disease development.

KBD-related experimental studies provide evidence that selenium deficiency and T-2 toxin exposure can induce osteochondral injury, with combined-exposure models supporting their joint effects on osteochondral tissues. Experimental studies also indicate that selenium deficiency or T-2 toxin exposure can be associated with alterations in gut microbial composition. However, whether these microbial changes mediate or modify exposure-related osteochondral injury remains unknown. Proposed pathways involving short-chain fatty acids, intestinal-barrier dysfunction, lipopolysaccharide-related inflammatory signaling, immune regulation, and bone remodeling are biologically plausible but are supported mainly by experimental evidence from non-KBD settings and have not been validated as an integrated causal pathway in KBD.

Accordingly, the microbiota–gut–osteochondral framework should be regarded as an evidence-stratified, hypothesis-generating model rather than an established KBD-specific mechanism. Future research should prioritize prospective studies in children and adolescents from endemic areas, together with appropriately designed experimental studies and direct measurement of proposed intermediate mediators, to clarify the temporal and causal relationships among environmental exposures, gut microbial alterations, and osteochondral injury. Such studies will help determine whether gut microbial alterations represent causal mediators, susceptibility modifiers, consequences, or correlates of KBD.

## Figures and Tables

**Table 1:** Evidence-stratified summary of key findings and limitations of studies relevant to Kashin–Beck disease.

Evidence Category	Study	Study Population/Model	Key Findings	Limitations
Human KBD	Han et al., 2025 [20]	Human KBD and control cartilage	Proteomic and transcriptomic analyses identified cartilage metabolic disturbances in KBD.	Small sample size; metabolic pathways inferred.
Human KBD	Ning et al., 2019 [21]	Juvenile and adult KBD patients	Alterations in NF-κB/RANKL-related signaling were identified.	No gut microbiota analysis.
Human KBD	Ning et al., 2022 [18]	KBD and knee OA patients	Gut microbiota composition differed between KBD and OA.	No healthy controls; cohort overlap with [17].
Human KBD	Wang et al., 2021 [17]	KBD patients and healthy controls	Gut dysbiosis was associated with altered serum metabolites.	Cross-sectional design.
Human KBD	Ning et al., 2023 [22]	Adult and pediatric KBD populations	Urinary metabolic alterations were identified in KBD.	Causality remains unclear.
Human KBD	Wen et al., 2023 [19]	KBD patients and healthy controls	Fecal metabolomics implicated altered selenium-related metabolic pathways.	Cross-sectional design.
KBD-related animal	Sun et al., 2021 [23]	Selenium-deficient and T-2-exposed rats	Selenium deficiency exacerbated T-2-induced cartilage injury.	No microbiota analysis.
KBD-related animal	Wu et al., 2023 [15]	Selenium-deficient or T-2-exposed rats	Selenium deficiency and T-2 exposure altered gut microbiota and cartilage pathology.	No microbiota mediation analysis.
Human–animal evidence	Yang et al., 2017 [7]	Human KBD cartilage and rat models	Selenium deficiency and T-2 exposure promoted chondrocyte apoptosis.	Small human sample size.
Human–animal evidence	Deng et al., 2024 [4]	Human cartilage and rat models	Low selenium and T-2 exposure affected AMPK/mTOR/ULK1-associated autophagy.	Limited human samples.
Human–animal evidence	Wu et al., 2025 [24]	Human KBD samples and rat models	Low selenium and T-2 exposure disrupted ZIP6-associated zinc homeostasis.	No microbiota analysis.
In vitro	Chen et al., 2023 [25]	IPEC-J2 intestinal epithelial cells	T-2 toxin impaired intestinal barrier function.	Non-KBD cell model.
In vitro	Yu et al., 2024 [6]	ATDC5 and rat cartilage cells	T-2 toxin induced mitochondrial dysfunction involving p53/CypD signaling.	Cell-model evidence.
Indirect evidence	AlQranei et al., 2021 [26]	Murine osteoclastogenesis model	LPS/TLR4 signaling promoted osteoclastogenesis.	Indirect relevance to KBD.
Indirect evidence	Kasaikina et al., 2011 [27]	Dietary selenium and microbiota mouse models	Dietary selenium influenced gut microbiota and host selenoproteome expression.	No cartilage outcomes.
Indirect evidence	Lucas et al., 2018 [11]	Mouse bone-loss models	Microbial short-chain fatty acids regulated bone mass and osteoclast activity.	Non-KBD models.

Abbreviations: KBD, Kashin–Beck disease; OA, osteoarthritis; NF-κB, nuclear factor kappa B; RANKL, receptor activator of nuclear factor kappa B ligand; T-2, T-2 toxin; AMPK, AMP-activated protein kinase; mTOR, mechanistic target of rapamycin; ULK1, Unc-51-like kinase 1; ZIP6, Zrt- and Irt-like protein 6; CypD, cyclophilin D; LPS, lipopolysaccharide; TLR4, Toll-like receptor 4.

## Data Availability

No new data were created or analyzed in this study. Data sharing is not applicable to this article.

## Abbreviations

The following abbreviations are used in this manuscript:

AMPK	AMP-activated protein kinase
CNKI	China National Knowledge Infrastructure
IL-1β	interleukin-1β
IL-6	interleukin-6
KBD	Kashin–Beck disease
LC-MS	liquid chromatography–mass spectrometry
LPS	lipopolysaccharide
MMP1	matrix metalloproteinase 1
MMP3	matrix metalloproteinase 3
MMP13	matrix metalloproteinase 13
mTOR	mechanistic target of rapamycin
NF-κB	nuclear factor kappa B
OA	osteoarthritis
OPG	osteoprotegerin
PBMCs	peripheral blood mononuclear cells
PICRUSt2	Phylogenetic Investigation of Communities by Reconstruction of Unobserved States 2
RANKL	receptor activator of nuclear factor-κB ligand
ROS	reactive oxygen species
RUNX2	runt-related transcription factor 2
SCFAs	short-chain fatty acids
SECISBP2	SECIS-binding protein 2
SIRT1	sirtuin 1
Th17	T helper 17 cell
TIMP1	tissue inhibitor of metalloproteinases 1
TLR4	Toll-like receptor 4
TNF-α	tumor necrosis factor-α
Treg	regulatory T cell
ULK1	Unc-51-like kinase 1
ZIP6	Zrt- and Irt-like protein 6

## Appendix A

**Table A1:** Study characteristics, comparators, exposure conditions, analytical methods, and measured and inferred outcomes of the included studies.

Study	Location	Study Characteristics	Comparator	Exposure	Samples	Method	Measured Outcomes	Inferred Outcomes
Han et al., 2025 [20]	China	6 KBD/6 controls (proteomics); 3/3 (RNA-seq); ages 54–75 vs. 57–71 years	Normal controls	—	Knee articular cartilage	Proteomics; RNA-seq	Proteins, transcripts	Metabolic pathways
Ning et al., 2019 [21]	Yongshou County, Shaanxi, China	Juvenile KBD/control: 12/12; adult: 20/12	Healthy controls	—	PBMCs; articular cartilage	Microarray; immunohistochemistry	Molecular expression	Signaling pathways
Ning et al., 2022 [18]	Shaanxi, China	32 KBD/32 OA; mean age 62 years	Knee OA patients	—	Feces	16S rDNA sequencing; shotgun metagenomics	Microbial composition	Functional profiles
Wang et al., 2021 [17]	Xunyi County, Shaanxi, China	32 KBD/35 controls; grades I–II	Healthy endemic-area controls	—	Feces; serum	16S rDNA sequencing; shotgun metagenomics; LC-MS metabolomics	Microbial taxa, genes, metabolites	Microbial functions
Ning et al., 2023 [22]	Shaanxi, China	Adults: 39 KBD/19 controls; children: 5 KBD, 25 suspected KBD, 123 controls	Healthy controls	—	Urine	LC-MS metabolomics	Urinary metabolites	Metabolic pathways
Wen et al., 2023 [19]	Linyou County, Shaanxi, China	46 KBD/29 controls; grades I–II; ages 58.99 ± 7.43 vs. 57.54 ± 8.22 years	Healthy endemic-area controls	—	Feces	UHPLC-MS metabolomics	Fecal metabolites	Metabolic pathways
Sun et al., 2021 [23]	China	40 Dark Agouti rats; 10/group; 10–12 weeks old	Se-sufficient controls	Se deficiency ± T-2 toxin	Cartilage	Histology; qPCR; protein analysis	Cartilage pathology, molecular markers	—
Wu et al., 2023 [15]	Shaanxi, China	24 Sprague–Dawley rats; 8/group; 3 weeks old	Normal controls	Se-deficient diet or T-2 toxin	Feces/gut contents; knee cartilage	16S rDNA sequencing; histology	Microbiota, cartilage pathology	—
Yang et al., 2017 [7]	China	Human: 5 KBD/5 controls; rats: 20 normal, 20 Se deficiency, 25 T-2, 25 Se deficiency + T-2	Healthy children; normal rats	Se deficiency; T-2 toxin	Hand phalange cartilage; rat cartilage	TUNEL; IHC; Western blot; RT-PCR	Apoptosis markers, molecular changes	—
Deng et al., 2024 [4]	Shaanxi, China	Human cartilage samples; 72 rats, 12/group; 3 weeks old	Non-KBD cartilage; normal rats	Low-Se diet ± T-2 toxin	Human and rat articular cartilage	TEM; histology; qRT-PCR; Western blot	Molecular markers	Autophagy pathway
Wu et al., 2025 [24]	Shaanxi, China	Human: 10 KBD/10 controls; rats: 32, 8/group	Non-KBD controls; normal rats	Low-Se diet; T-2; low-Se + T-2 combined exposure	Human/rat articular cartilage and chondrocytes	Transcriptome sequencing; IHC; qRT-PCR; Western blot	Molecular outcomes	Transcriptomic pathways
Chen et al., 2023 [25]	China	IPEC-J2 intestinal epithelial cells	Untreated/vehicle controls	T-2 toxin	IPEC-J2 intestinal epithelial cells	Barrier assays; oxidative stress assays; molecular analysis	Cellular outcomes	Signaling pathways
Yu et al., 2024 [6]	China	ATDC5 and rat cartilage cells	Vehicle controls	T-2 toxin	ATDC5 chondrocytes	Transcriptomics; TEM; mitochondrial assays; protein analysis	Cellular outcomes	Pathway targets
AlQranei et al., 2021 [26]	United States	Murine osteoclast models	RANKL-only/control conditions	LPS/TLR4 signaling	Osteoclast lineage cells	Osteoclast assays; molecular analysis	Cellular outcomes	Signaling pathways
Kasaikina et al., 2011 [27]	United States	Dietary Se and germ-free/conventionalized mouse models	Se-sufficient mice; germ-free vs. colonized mice	Dietary selenium	Gut contents; host tissues	Microbiota sequencing; molecular analysis	Microbiota, host responses	—
Lucas et al., 2018 [11]	Germany	Mouse bone-loss models	Untreated/control groups	Propionate and butyrate (SCFAs)	Bone, marrow and systemic samples	Micro-CT; histomorphometry; molecular analysis	Bone outcomes	SCFA effects

Abbreviations: KBD, Kashin–Beck disease; OA, osteoarthritis; RNA-seq, RNA sequencing; PBMCs, peripheral blood mononuclear cells; rDNA, ribosomal deoxyribonucleic acid; LC-MS, liquid chromatography–mass spectrometry; UHPLC-MS, ultra-high-performance liquid chromatography–mass spectrometry; Se, selenium; T-2, T-2 toxin; qPCR, quantitative polymerase chain reaction; TUNEL, terminal deoxynucleotidyl transferase-mediated dUTP nick-end labeling; IHC, immunohistochemistry; RT-PCR, reverse transcription polymerase chain reaction; TEM, transmission electron microscopy; qRT-PCR, quantitative reverse transcription polymerase chain reaction; IPEC-J2, intestinal porcine epithelial cell line J2; ATDC5, ATDC5 chondrogenic cell line; RANKL, receptor activator of nuclear factor kappa B ligand; LPS, lipopolysaccharide; TLR4, Toll-like receptor 4; SCFAs, short-chain fatty acids; micro-CT, micro-computed tomography.

## References

[1] Guo X., Ma W.J., Zhang F., Ren F.L., Qu C.J., Lammi M.J. (2014). Recent advances in the research of an endemic osteochondropathy in China: Kashin-Beck disease. Osteoarthr. Cartil..

[2] Cui S.L., Liu H., Pei J.R., Li J.X., Jiao Z., Deng Q., Liu N., Cao Y.H., Yu J. (2024). Summary Analysis of National Surveillance on Kashin-Beck Disease from 1990 to 2023. Biomed. Environ. Sci..

[3] Liu L., Luo P., Wen P., Xu P. (2024). Effects of selenium and iodine on Kashin-Beck disease: An updated review. Front. Nutr..

[4] Deng H., Lin X., Xiang R., Bao M., Qiao L., Liu H., He H., Wen X., Han J. (2024). Low selenium and T-2 toxin may be involved in the pathogenesis of Kashin-Beck disease by affecting AMPK/mTOR/ULK1 pathway mediated autophagy. Ecotoxicol. Environ. Saf..

[5] Chen J., Xue S., Li S., Wang Z.-L., Yang H., Wang W., Song D., Zhou X.R., Chen C. (2012). Oxidant damage in Kashin-Beck disease and a rat Kashin-Beck disease model by employing T-2 toxin treatment under selenium deficient conditions. J. Orthop. Res..

[6] Yu F.F., Yu S.Y., Sun L., Zuo J., Luo K.T., Wang M., Fu X.L., Zhang F., Huang H., Zhou G.Y. (2024). T-2 toxin induces mitochondrial dysfunction in chondrocytes via the p53-cyclophilin D pathway. J. Hazard Mater..

[7] Yang H.J., Zhang Y., Wang Z.L., Xue S.H., Li S.Y., Zhou X.R., Zhang M., Fang Q., Wang W.J., Chen C. (2017). Increased Chondrocyte Apoptosis in Kashin-Beck Disease and Rats Induced by T-2 Toxin and Selenium Deficiency. Biomed. Environ. Sci. BES.

[8] Chen J., Chu Y., Cao J., Wang W., Liu J., Wang J. (2011). Effects of T-2 toxin and selenium on chondrocyte expression of matrix metalloproteinases (MMP-1, MMP-13), α2-macroglobulin (α2M) and TIMPs. Toxicol. Vitr..

[9] Thursby E., Juge N. (2017). Introduction to the human gut microbiota. Biochem. J..

[10] Zaiss M.M., Jones R.M., Schett G., Pacifici R. (2019). The gut-bone axis: How bacterial metabolites bridge the distance. J. Clin. Investig..

[11] Lucas S., Omata Y., Hofmann J., Böttcher M., Iljazovic A., Sarter K., Albrecht O., Schulz O., Krishnacoumar B., Krönke G. (2018). Short-chain fatty acids regulate systemic bone mass and protect from pathological bone loss. Nat. Commun..

[12] Ramires L.C., Santos G.S., Ramires R.P., da Fonseca L.F., Jeyaraman M., Muthu S., Lana A.V., Azzini G., Smith C.S., Lana J.F. (2022). The Association between Gut Microbiota and Osteoarthritis: Does the Disease Begin in the Gut?. Int. J. Mol. Sci..

[13] Longo U.G., Lalli A., Bandini B., de Sire R., Angeletti S., Lustig S., Ammendolia A., Budhiparama N.C., de Sire A. (2024). Role of the Gut Microbiota in Osteoarthritis, Rheumatoid Arthritis, and Spondylarthritis: An Update on the Gut-Joint Axis. Int. J. Mol. Sci..

[14] Li S., Mao Y., Zhou F., Yang H., Shi Q., Meng B. (2020). Gut microbiome and osteoporosis: A review. Bone Jt. Res..

[15] Wu Y., Gong Y., Zhang Y., Li S., Wang C., Yuan Y., Lv X., Liu Y., Chen F., Chen S. (2023). Comparative Analysis of Gut Microbiota from Rats Induced by Se Deficiency and T-2 Toxin. Nutrients.

[16] Liu L., Bai L., Wu Y., Zhang Y., Wang C., Li S., Yuan Y., Lv X., Wang H., Qin Y. (2025). Exploring the role of gut microbiota in the pathogenesis of Kashin-Beck Disease: A Focus on selenium deficiency and T-2 toxin exposure. Med. Hypotheses.

[17] Wang X., Ning Y., Li C., Gong Y., Huang R., Hu M., Poulet B., Xu K., Zhao G., Zhou R. (2021). Alterations in the gut microbiota and metabolite profiles of patients with Kashin-Beck disease, an endemic osteoarthritis in China. Cell Death Dis..

[18] Ning Y., Hu M., Gong Y., Huang R., Xu K., Chen S., Zhang F., Liu Y., Chen F., Chang Y. (2022). Comparative analysis of the gut microbiota composition between knee osteoarthritis and Kashin-Beck disease in Northwest China. Arthritis Res. Ther..

[19] Wen Y., Wang B., Shi P., Chu X., Shi S., Yao Y., Zhang L., Zhang F. (2023). A Metabolomics Study of Feces Revealed That a Disturbance of Selenium-Centered Metabolic Bioprocess Was Involved in Kashin-Beck Disease, an Osteoarthropathy Endemic to China. Nutrients.

[20] Han L., Cheng B., Xia J., Cheng S., Yang X., Zhang F. (2025). Integrative Proteome and Transcriptome Analyses Reveal the Metabolic Disturbance of the Articular Cartilage in Kashin-Beck Disease, an Endemic Arthritis. Int. J. Mol. Sci..

[21] Ning Y., Wang X., Lammi M.J., Guo X. (2019). Changes in the NF-κB signaling pathway in juvenile and adult patients with Kashin-Beck disease. Exp. Cell Res..

[22] Ning Y., Chen S., Zhang F., Liu Y., Chen F., Li S., Wang C., Wu Y., Gong Y., Hu M. (2023). The alteration of urinary metabolomics profiles in Kashin-Beck disease in a three consecutive year study. Mol. Omics.

[23] Sun J., Min Z.X., Zhao W.X., Hussain S., Zhao Y.T., Guo D.X., Zhang F.J., Guo Y.X., Sun M.Y., Huang H. (2021). T-2 Toxin Induces Epiphyseal Plate Lesions via Decreased SECISBP2-Mediated Selenoprotein Expression in DA Rats, Exacerbated by Selenium Deficiency. CARTILAGE.

[24] Wu Y., Gong Y., Liu L., Bai L., Zhang Y., Li S., Wang C., Yuan Y., Lv X., Qin Y. (2025). The Impact of Selenium Deficiency and T-2 Toxin on Zip6 Expression in Kashin-Beck Disease. Biol. Trace Elem. Res..

[25] Chen F., Wang Y., Chen Y., Fan J., Zhang C., He X., Yang X. (2023). JNK molecule is a toxic target for IPEC-J2 cell barrier damage induced by T-2 toxin. Ecotoxicol. Environ. Saf..

[26] AlQranei M.S., Senbanjo L.T., Aljohani H., Hamza T., Chellaiah M.A. (2021). Lipopolysaccharide- TLR-4 Axis regulates Osteoclastogenesis independent of RANKL/RANK signaling. BMC Immunol..

[27] Kasaikina M.V., Kravtsova M.A., Lee B.C., Seravalli J., Peterson D.A., Walter J., Legge R., Benson A.K., Hatfield D.L., Gladyshev V.N. (2011). Dietary selenium affects host selenoproteome expression by influencing the gut microbiota. FASEB J..

[28] Zhang Y., Roh Y.J., Han S.J., Park I., Lee H.M., Ok Y.S., Lee B.C., Lee S.R. (2020). Role of Selenoproteins in Redox Regulation of Signaling and the Antioxidant System: A Review. Antioxidants.

[29] Ye R., Huang J., Wang Z., Chen Y., Dong Y. (2022). The Role and Mechanism of Essential Selenoproteins for Homeostasis. Antioxidants.

[30] Minich W.B. (2022). Selenium Metabolism and Biosynthesis of Selenoproteins in the Human Body. Biochemistry.

[31] Zhang F., Li X., Wei Y. (2023). Selenium and Selenoproteins in Health. Biomolecules.

[32] Yang X., Li Z., Zhang R., Zhang D., Xiong Y., Wang C., Yang X., Li Q. (2022). Dysregulation of Transcription Profile of Selenoprotein in Patients with Kashin-Beck Disease and Its Effect on Se Deficiency-Induced Chondrocyte Apoptosis. Biol. Trace Elem. Res..

[33] Wang W., Wei S., Luo M., Yu B., Cao J., Yang Z., Wang Z., Goldring M.B., Chen J. (2013). Oxidative stress and status of antioxidant enzymes in children with Kashin-Beck disease. Osteoarthr. Cartil..

[34] Moreno-Reyes R., Suetens C., Mathieu F., Begaux F., Zhu D., Rivera M.T., Boelaert M., Nève J., Perlmutter N., Vanderpas J. (1998). Kashin-Beck osteoarthropathy in rural Tibet in relation to selenium and iodine status. N. Engl. J. Med..

[35] Chen Z., Li H., Yang L., Wang W., Li Y., Gong H., Guo M., Nima C., Zhao S., Wang J. (2015). Hair Selenium Levels of School Children in Kashin-Beck Disease Endemic Areas in Tibet, China. Biol. Trace Elem. Res..

[36] Zhang S., Xing Z., Du H., Qi F., Yu J. (2026). Investigation of Selenium and T-2 Toxin Exposure Levels in the Internal and External Environments of Key Areas of Kashin Beck Disease in China. Biol. Trace Elem. Res..

[37] Zhang D., Li Q., Yang X., Xiong Y., Sun S. (2025). Effects of selenium-mediated RUNX2 overexpression and its transcriptome alterations on Chondrocyte injury in Kashin Beck disease. Front. Cell Dev. Biol..

[38] Hondal R.J. (2023). Selenium vitaminology: The connection between selenium, vitamin C, vitamin E, and ergothioneine. Curr. Opin. Chem. Biol..

[39] Aaseth J.O., Finnes T.E., Askim M., Alexander J. (2024). The Importance of Vitamin K and the Combination of Vitamins K and D for Calcium Metabolism and Bone Health: A Review. Nutrients.

[40] Rooze S., Mathieu F., Claus W., Yangzom T., Yangzom D., Goyens P., de Maertelaer V. (2016). Effect of calcium and vitamin D on growth, rickets and Kashin-Beck disease in 0- to 5-year-old children in a rural area of central Tibet. Trop. Med. Int. Health.

[41] Wang K., Yu J., Liu H., Liu Y., Liu N., Cao Y., Zhang X., Sun D. (2020). Endemic Kashin-Beck disease: A food-sourced osteoarthropathy. Semin. Arthritis Rheum..

[42] Xue N., Zhao J., Yin J., Liu L., Yang Z., Zhai S., Bian X., Gao X. (2025). The Role of SUMO1 Modification of SOX9 in Cartilage Development Stimulated by Zinc Ions in Mice. Organogenesis.

[43] O’Connor J.P., Kanjilal D., Teitelbaum M., Lin S.S., Cottrell J.A. (2020). Zinc as a Therapeutic Agent in Bone Regeneration. Materials.

[44] Zha X., An J., Gao X., Tian Y. (2022). Dietary and drinking water intake of essential trace elements in a typical Kashin-Beck disease endemic area of Tibet, China. Environ. Health A Glob. Access Sci. Source.

[45] Guo Y., Li H., Yang L., Li Y., Wei B., Wang W., Gong H., Guo M., Nima C., Zhao S. (2017). Trace Element Levels in Scalp Hair of School Children in Shigatse, Tibet, an Endemic Area for Kaschin-Beck Disease (KBD). Biol. Trace Elem. Res..

[46] Zhang F., Wu C., Zhang P., Wang X., Meng P., Tan S., Yuan L., Guo X. (2022). Abnormal Level of Manganese, Iron, Iodine, and Selenium in the Hair of Children Living in Kashin-Beck Disease Endemic Areas. Biol. Trace Elem. Res..

[47] Fu G., Chen X., Qi M., Du X., Xia Z., Liu Q., Sun N., Shi C., Zhang R. (2022). Status and potential diagnostic roles of essential trace elements in Kashin- Beck disease patients. J. Trace Elem. Med. Biol..

[48] Jing X., Du T., Li T., Yang X., Wang G., Liu X., Jiang Z., Cui X. (2021). The detrimental effect of iron on OA chondrocytes: Importance of pro-inflammatory cytokines induced iron influx and oxidative stress. J. Cell. Mol. Med..

[49] Janik E., Niemcewicz M., Podogrocki M., Ceremuga M., Stela M., Bijak M. (2021). T-2 Toxin-The Most Toxic Trichothecene Mycotoxin: Metabolism, Toxicity, and Decontamination Strategies. Molecules.

[50] Yin H., Han S., Chen Y., Wang Y., Li D., Zhu Q. (2020). T-2 Toxin Induces Oxidative Stress, Apoptosis and Cytoprotective Autophagy in Chicken Hepatocytes. Toxins.

[51] Dai C., Xiao X., Sun F., Zhang Y., Hoyer D., Shen J., Tang S., Velkov T. (2019). T-2 toxin neurotoxicity: Role of oxidative stress and mitochondrial dysfunction. Arch. Toxicol..

[52] He W., Wang J., Han M., Wang L., Li L., Zhang J., Chen S., Guo J., Zhai X., Yang J. (2023). Potential Toxicity and Mechanisms of T-2 and HT-2 Individually or in Combination on the Intestinal Barrier Function of Porcine Small Intestinal Epithelial Cells. Toxins.

[53] Pomothy J.M., Szabó O., Czimmermann Á.E., Babiczky Á., Jerzsele Á., Pászti-Gere E. (2021). Investigation of the inflammatory and oxidative stress-inducing effects of deoxynivalenol and T-2 toxin exposure in non-tumorigenic human intestinal cell model. Toxicon.

[54] Lin R., Sun Y., Ye W., Zheng T., Wen J., Deng Y. (2019). T-2 toxin inhibits the production of mucin via activating the IRE1/XBP1 pathway. Toxicology.

[55] Liu M., Li X.W., Sun H., Yan Y.Q., Xia Z.Y., Refaie A., Zhang N.Y., Wang S., Tan C., Sun L.H. (2025). T-2 toxin-induced splenic injury by disrupting the gut microbiota-spleen axis via promoting IL-6/JAK/STAT1 signaling-mediated inflammation and apoptosis and its mitigation by elemental nano-selenium. Arch. Toxicol..

[56] Guan F., Li S., Wang Z.-L., Yang H., Xue S., Wang W., Song D., Zhou X., Zhou W., Chen J.-H. (2013). Histopathology of chondronecrosis development in knee articular cartilage in a rat model of Kashin–Beck disease using T-2 toxin and selenium deficiency conditions. Rheumatol. Int..

[57] Zhou X., Yang H., Guan F., Xue S., Song D., Chen J., Wang Z. (2016). T-2 Toxin Alters the Levels of Collagen II and Its Regulatory Enzymes MMPs/TIMP-1 in a Low-Selenium Rat Model of Kashin-Beck Disease. Biol. Trace Elem. Res..

[58] Yang X., Han L., Zhang D., Xu C., Jin Z., Xiong Y. (2025). Downregulated Expression and Hypermethylation of SIRT1 in Patients with Kashin-Beck Disease-Mediated Chondrocyte Apoptosis May Potentially Be Ameliorated by Selenium Supplement. Biol. Trace Elem. Res..

[59] Ghosh T.S., Shanahan F., O’Toole P.W. (2022). The gut microbiome as a modulator of healthy ageing. Nat. Rev. Gastroenterol. Hepatol..

[60] Badal V.D., Vaccariello E.D., Murray E.R., Yu K.E., Knight R., Jeste D.V., Nguyen T.T. (2020). The Gut Microbiome, Aging, and Longevity: A Systematic Review. Nutrients.

[61] Huang Q., Zhou Z.K., Ma J., Li Y., Yang X., Shen B., Yang J., Kang P.D., Pei F.X. (2015). The arthropathic and functional impairment features of adult Kashin-Beck disease patients in Aba Tibetan area in China. Osteoarthr. Cartil..

[62] Wang W., Kou F., Wang J., Quan Z., Zhao S., Wang Y., Hu X., Sun H., Cao L. (2022). Pretreatment with millet-derived selenylated soluble dietary fiber ameliorates dextran sulfate sodium-induced colitis in mice by regulating inflammation and maintaining gut microbiota balance. Front. Nutr..

[63] Zhao M., Xia P., Zhang X., He H., Hou T. (2023). Selenium-containing soybean peptides ameliorate intestinal inflammation and modulate gut microbiota dysbacteriosis in DSS-induced ulcerative colitis mice. Food Funct..

[64] Koh A., De Vadder F., Kovatcheva-Datchary P., Bäckhed F. (2016). From Dietary Fiber to Host Physiology: Short-Chain Fatty Acids as Key Bacterial Metabolites. Cell.

[65] Tan J., McKenzie C., Potamitis M., Thorburn A.N., Mackay C.R., Macia L. (2014). The role of short-chain fatty acids in health and disease. Adv. Immunol..

[66] Jin D., Wu S., Zhang Y.G., Lu R., Xia Y., Dong H., Sun J. (2015). Lack of Vitamin D Receptor Causes Dysbiosis and Changes the Functions of the Murine Intestinal Microbiome. Clin. Ther..

[67] Singh P., Rawat A., Alwakeel M., Sharif E., Al Khodor S. (2020). The potential role of vitamin D supplementation as a gut microbiota modifier in healthy individuals. Sci. Rep..

[68] Charoenngam N., Shirvani A., Kalajian T.A., Song A., Holick M.F. (2020). The Effect of Various Doses of Oral Vitamin D_3_ Supplementation on Gut Microbiota in Healthy Adults: A Randomized, Double-blinded, Dose-response Study. Anticancer Res..

[69] Wyatt M., Choudhury A., Von Dohlen G., Heileson J.L., Forsse J.S., Rajakaruna S., Zec M., Tfaily M.M., Greathouse L. (2024). Randomized control trial of moderate dose vitamin D alters microbiota stability and metabolite networks in healthy adults. Microbiol. Spectr..

[70] Li J., Ho W.T.P., Liu C., Chow S.K., Ip M., Yu J., Wong H.S., Cheung W.H., Sung J.J.Y., Wong R.M.Y. (2021). The role of gut microbiota in bone homeostasis. Bone Jt. Res..

[71] Tu Y., Yang R., Xu X., Zhou X. (2021). The microbiota-gut-bone axis and bone health. J. Leukoc. Biol..

[72] Tyagi A.M., Yu M., Darby T.M., Vaccaro C., Li J.Y., Owens J.A., Hsu E., Adams J., Weitzmann M.N., Jones R.M. (2018). The Microbial Metabolite Butyrate Stimulates Bone Formation via T Regulatory Cell-Mediated Regulation of WNT10B Expression. Immunity.

[73] Peng L., Li Z.R., Green R.S., Holzman I.R., Lin J. (2009). Butyrate enhances the intestinal barrier by facilitating tight junction assembly via activation of AMP-activated protein kinase in Caco-2 cell monolayers. J. Nutr..

[74] Ghosh S.S., Wang J., Yannie P.J., Ghosh S. (2020). Intestinal Barrier Dysfunction, LPS Translocation, and Disease Development. J. Endocr. Soc..

[75] Lu Y.C., Yeh W.C., Ohashi P.S. (2008). LPS/TLR4 signal transduction pathway. Cytokine.

[76] Mazgaeen L., Gurung P. (2020). Recent Advances in Lipopolysaccharide Recognition Systems. Int. J. Mol. Sci..

[77] Yan D., Pei F., Song Y. (2014). Serum levels of M-CSF, RANKL and OPG in rats fed with Kashin-Beck disease-affected diet. J. Orthop. Surg. Res..

[78] Amarasekara D.S., Kim S., Rho J. (2021). Regulation of Osteoblast Differentiation by Cytokine Networks. Int. J. Mol. Sci..

[79] Zhu L., Hua F., Ding W., Ding K., Zhang Y., Xu C. (2020). The correlation between the Th17/Treg cell balance and bone health. Immun. Ageing.

[80] Zhang Y., He Y., Zhang D., Zhang M., Wang M., Zhang Y., Ma T., Chen J. (2018). Death of chondrocytes in Kashin-Beck disease: Apoptosis, necrosis or necroptosis?. Int. J. Exp. Pathol..

[81] Yatsunenko T., Rey F.E., Manary M.J., Trehan I., Dominguez-Bello M.G., Contreras M., Magris M., Hidalgo G., Baldassano R.N., Anokhin A.P. (2012). Human gut microbiome viewed across age and geography. Nature.

[82] Stewart C.J., Ajami N.J., O’Brien J.L., Hutchinson D.S., Smith D.P., Wong M.C., Ross M.C., Lloyd R.E., Doddapaneni H., Metcalf G.A. (2018). Temporal development of the gut microbiome in early childhood from the TEDDY study. Nature.

[83] Rayman M.P. (2012). Selenium and human health. Lancet.

